# Computational Study of Ternary Devices: Stable, Low-Cost, and Efficient Planar Perovskite Solar Cells

**DOI:** 10.1007/s40820-018-0205-5

**Published:** 2018-05-17

**Authors:** Sajid Sajid, Ahmed Mourtada Elseman, Jun Ji, Shangyi Dou, Dong Wei, Hao Huang, Peng Cui, Wenkang Xi, Lihua Chu, Yingfeng Li, Bing Jiang, Meicheng Li

**Affiliations:** 1 State Key Laboratory of Alternate Electrical Power, System with Renewable Energy Sources, School of Renewable Energy, North China Electric Power University, Beijing, 102206 People’s Republic of China; 2 Electronic and Magnetic Materials Department, Advanced Materials Division, Central Metallurgical Research and Development Institute (CMRDI), P.O. Box 87, Helwan, Cairo, 11421 Egypt

**Keywords:** Perovskite solar cells, Copper-doped nickel oxide, Zinc oxide, Simulation

## Abstract

Although perovskite solar cells with power conversion efficiencies (PCEs) more than 22% have been realized with expensive organic charge-transporting materials, their stability and high cost remain to be addressed. In this work, the perovskite configuration of MAPbX (MA = CH_3_NH_3_, X = I_3_, Br_3_, or I_2_Br) integrated with stable and low-cost Cu:NiO_*x*_ hole-transporting material, ZnO electron-transporting material, and Al counter electrode was modeled as a planar PSC and studied theoretically. A solar cell simulation program (wxAMPS), which served as an update of the popular solar cell simulation tool (AMPS: Analysis of Microelectronic and Photonic Structures), was used. The study yielded a detailed understanding of the role of each component in the solar cell and its effect on the photovoltaic parameters as a whole. The bandgap of active materials and operating temperature of the modeled solar cell were shown to influence the solar cell performance in a significant way. Further, the simulation results reveal a strong dependence of photovoltaic parameters on the thickness and defect density of the light-absorbing layers. Under moderate simulation conditions, the MAPbBr_3_ and MAPbI_2_Br cells recorded the highest PCEs of 20.58 and 19.08%, respectively, while MAPbI_3_ cell gave a value of 16.14%.

**Electronic supplementary material:**

The online version of this article (10.1007/s40820-018-0205-5) contains supplementary material, which is available to authorized users.

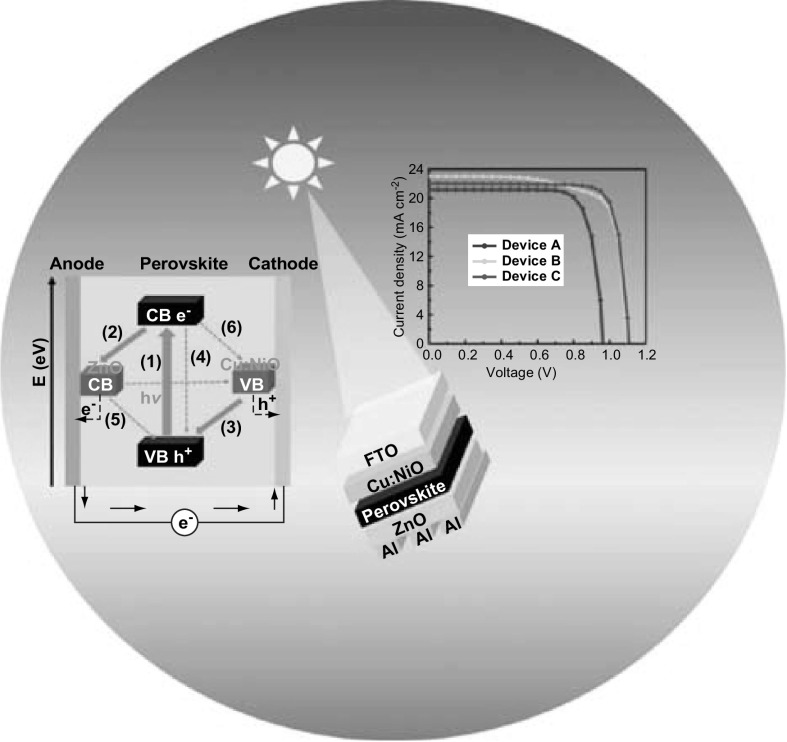

## Highlights

- Simulation study of perovskite solar cells with *p*–*i*–*n* structure in the configuration Cu:NiO_*x*_/MAPbX/ZnO/Al.
- Solar cells with MAPbI_2_Br photoactive layers exhibit best conversion efficiency and show promise of high thermal stability.
- Simulation results provide a better understanding of the defect density and thickness of the active perovskite layer.

## Introduction

Since the emergence of organic–inorganic halide perovskite materials as efficient light harvesters, perovskite solar cells (PSCs) have been widely investigated in terms of their power conversion efficiencies (PCEs). Fundamental studies on perovskite materials [1, 2], device architectures [3–5], fabrication processes [6, 7], and material engineering [8, 9] have assisted the rapid development of PSCs. Consequently, PCEs greater than 22% have been achieved, as certified by National Renewable Energy Laboratory (NREL) [10]. Presently, the PSC is considered as one of the most promising alternative energy conversion devices in terms of high efficiency and low cost. However, despite the major achievements in scaling efficiency levels in the laboratory ambient, the concern of long-term stability and performance of these solar cells under severe humidity and light illumination conditions as well as the requirement of expensive organic charge-transporting materials (OCTMs) remain critical. In order to achieve high performance and improved stability, several material refinements have been attempted by substituting a mixture of suitable materials in the organic and halide sites (organic site: MA, HC(NH_2_)_2_; halide site: I, Br, Cl) [8, 9, 11–15]. Despite their application in most efficient devices, the expensive OCTMs are limited in use by a tendency to absorb water, difficulty in synthesizing, and inability to manage charge carriers effectively. Use of *p*-type and *n*-type inorganic materials would be a long-term solution for constructing stable PSCs more economically [16–19]. In this context, metal oxide semiconductors are considered chemically stable and highly transparent with tunable energy band levels [20–24]. They are nontoxic and abundantly available in the earth’s crust for universal use as photovoltaic materials. More attractively, thin layers of these materials have been deposited using low-cost techniques such as spin coating, doctor blading, and spray pyrolysis in ambient atmosphere. However, low charge carrier mobility and fill factor (FF) associated with inorganic HTMs, particularly by the use of NiO_*x*_ and Cu_2_O, are deterrents compared to organic HTMs. Highly efficient planar PSCs incorporated with solution-processed copper-doped NiO_*x*_ as HTMs have been demonstrated by many researchers [25–27]. These studies revealed that the high conductivities (of HTM/ETM) obtained by doping or managing the charge carrier collection and valence/conduction band matching with perovskite absorber have a positive impact on PCE [6, 26, 28, 29]. In this work, we engaged a one-dimensional software program (wxAMPS), which is widely used for simulating carrier transport in solid-state devices, to derive continuity and Poisson equations from first principles and to analyze the transport behavior of perovskite device structures. From the simulation output, solar cell parameters such as current–voltage characteristics in the dark and, if desired, under illumination can be obtained. For numerical simulation of PSCs such as the TiO_2_/MAPbI_3_/spiro-OMeTAD/Au, an effective method has been described by Liu et al. [30], which helps in identifying the optimal operating conditions.

In this context, we were inspired by Cu:NiO_*x*_ as a hole-transporting layer (HTL) and ZnO as electron-transporting layer (ETL), which are highly conducting, environmental-friendly, and economically fabricable materials, together with Al back contact for integrating MAPbI_3_, MAPbI_2_Br, and MAPbBr_3_ thin layers to a *p*–*i*–*n* configuration. It was required to understand the role of each component and their combined effect so as to achieve incremental improvement in the photovoltaic characteristics of the PSC thus designed. In this endeavor, a theoretical study was performed to identify the optimal conditions of device operation using the simulation software wxAMPS and by employing intra-band and trap-assisted tunneling models for heterojunction analysis. This study represents the first (in our knowledge) numerical simulation of a PSC based on the perovskite series MAPbX (MA = CH_3_NH_3_, X = I_3_, Br_3_, or I_2_Br) in conjunction with Cu:NiO_*x*_ (HTL), ZnO (ETL), and Al counter electrode and is aimed at improving the design of PSCs which are highly efficient, stable, and economical to fabricate.

## Modeling of Perovskite Solar Cell

**Fig. 1:**
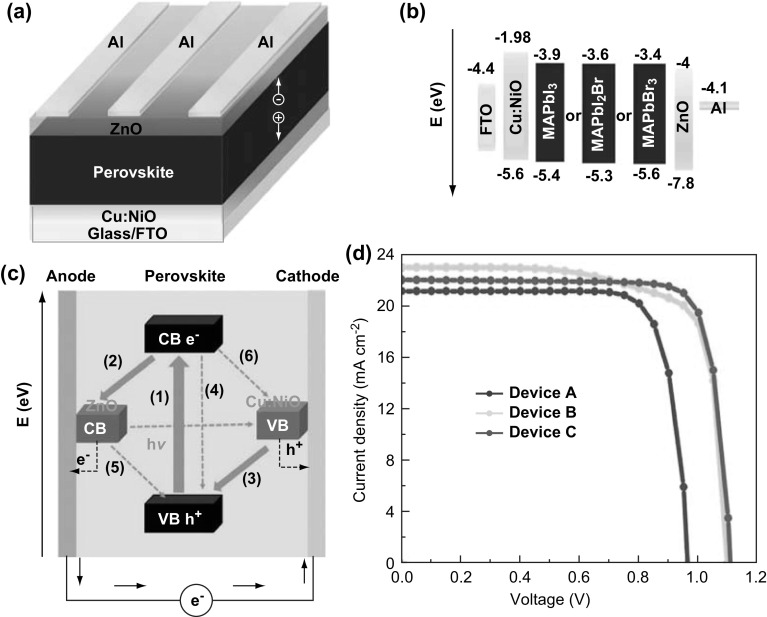
**a** Schematic crosssection of *p*–*i*–*n* perovskite solar cells. **b** Energy band diagram of the corresponding heterojunctions. **c** Charge generation, separation, and extraction processes. **d** Simulated *J*–*V* characteristic curves under AM1.5G illumination at 100 mW cm^−2^ irradiance

In the simulation process, device models were constructed with thin film stacks of glass/FTO/Cu:NiO_*x*_/MAPbX/ZnO/Al (MA = CH_3_NH_3_, X = I_3_, Br_3_, or I_2_Br) which are illustrated in Fig. 1a, and the corresponding energy band diagrams are shown in Fig. 1b. The paths of generation and recombination of charge carriers in PSCs are shown in Fig. 1c. The charge generation, separation, and extraction processes shown as steps (1), (2), and (3), respectively, are dynamic processes that are favorable for high-performance PSCs. In order to manage the charge collection efficiently, these processes should occur at a much faster rate than that of the recombination process (undesirable), which are shown in steps (4)–(6). For any further optimization and enhancement in photovoltaic parameters, one needs to address the timescale and charge carrier kinetics occurring at different interfaces in PSCs. In this context, three device layouts were created and labeled as devices A, B, and C using MAPbI_3_, MAPbI_2_Br, and MAPbBr_3_ active layers, respectively. The AM1.5G solar spectrum was adopted as the illumination source for the simulation. The light reflectance at the top and bottom contacts was set to 0 and 1, respectively. The material parameters used for the simulation are summarized in Table 1; these were carefully chosen from earlier experimental reports [14, 23, 31]. Capture cross sections of both electrons and holes were set to 1 × 10^−16^ cm^2^ which resulted in a carrier diffusion length of ~ 1 μm in light-absorbing materials. The thicknesses of the active layer were set to 0.4 μm for device A and 0.5 μm for devices B and C, respectively, for efficient charge carrier collection. The possibility of Gaussian states that are either donor-like or acceptor-like and which are located anywhere in the bandgap can be predicted by wxAMPS. The donor-like tail states coming out of the valence band can be modeled in wxAMPS by Eq. 1,

1 $$g_{\text{d}} (E_{1} ) = G_{{{\text{d}}0}} { \exp }^{{\left( {{\raise0.7ex\hbox{${ - E_{1} }$} \!\mathord{\left/ {\vphantom {{ - E_{1} } {E_{\text{d}} }}}\right.\kern-0pt} \!\lower0.7ex\hbox{${E_{\text{d}} }$}}} \right)}}$$

where *g*_d_ is donor-like tail Gaussian state, *E*_1_ is measured positively from the valence band edge (*E*_v_) upward to the point located at *x*, *E*_d_ is the characteristic energy that determines the slope of the tail, and *G*_d0_ represents the density of states. Similarly, the acceptor-like tail states coming out of the conduction band can be modeled by Eq. 2,

2 $$g_{\text{a}} (E_{2} ) = G_{{{\text{a}}0}} { \exp }^{{\left( {{\raise0.7ex\hbox{${ - E_{2} }$} \!\mathord{\left/ {\vphantom {{ - E_{2} } {E_{\text{a}} }}}\right.\kern-0pt} \!\lower0.7ex\hbox{${E_{\text{a}} }$}}} \right)}} ,$$

where g_a_ is acceptor-like tail Gaussian state, *E*_2_ is measured negatively from the conduction band edge (*E*_c_) downward to the same point *x*, *E*_a_ is the characteristic energy that determines the slope of the tail, and *G*_a0_ represents the density of states.

**Table 1:** Basic material parameters used for the simulation, which were carefully chosen from experimental reports [14, 23, 31]

Material	$\varepsilon$	*E*_g_	Affinity	*N*_c_ (conduction band)	*N*_v_ (valence band)	*μ*_e_ (cm^2^/Vs)	*μ*_h_ (cm^2^/Vs)	*N*_d_ (donor conc.) cm^−3^	*N*_a_ (acceptor conc.) cm^−3^	References
MAPbI_3_	10	1.5	3.93	2.8 × 10^18^	3.9 × 10^18^	10	10	1 × 10^9^	1 × 10^9^	[30, 34, 35]
MAPbI_2_Br	15	1.7	3.77	3 × 10^18^	4.0 × 10^18^	15	15	1 × 10^10^	1 × 10^10^	[14]
MAPbBr_3_	17	2.2	3.38	3.5 × 10^18^	4.0 × 10^19^	17	17	2.14 × 10^17^	2.14 × 10^17^	[14, 36]
ZnO	9	3.3	4.40	2.2 × 10^18^	1.8 × 10^19^	10	2.5	1 × 10^18^	0.0	[37]
Cu:NiO	11	3.62	2.0	1.6 × 10^18^	2.9 × 10^20^	1.4	4.9	0.0	1.5 × 10^18^	[26]

According to Eqs. 1 and 2, the defect energy levels in the simulated thin film materials are located at the center of the bandgap with the Gaussian-type energy distribution (characteristic energy of 0.1 eV). The tail characteristic energy is 0.01 eV with density of band-tail states of 1 × 10^14^ cm^−3^ eV^−1^ [30, 32, 33].

Since the performance of the solar cell is influenced by bimolecular electron–hole recombination rate, two assumptions have been made to understand the bimolecular recombination rate. In the first assumption, the conduction band electrons are directed to empty states in the valence band, a process known as band-to-band or direct recombination *R*_D_ (also called intrinsic recombination). In the second assumption, electrons and holes recombine through intermediate gap states known as recombination centers or by a process of extrinsic recombination. The latter is labeled as indirect recombination *R*_I_ or Shockley–Read–Hall recombination. The total recombination term *R*(*x*) in the continuity equation takes into account both these processes as a sum of the two components (Eq. 3).

3 $$R\left( x \right) \, = \, R_{\text{D}} \left( x \right) \, + \, R_{\text{I}} \left( x \right)$$

Additionally, the bimolecular recombination rate simply relies on an overlap of electron and hole wavefunctions, while Auger processes involve transfer of energy and momentum of the recombining electron–hole pair to a third charge carrier. On the other hand, experimental results of THz photoconductivity transients for perovskite materials have been documented [34] which lead to bimolecular and monomolecular charge recombination rates according to Eq. 4,

4 $$\frac{{{\text{d}}n}}{{{\text{d}}t}} = - \gamma np - \gamma np_{0}$$

where *n* denotes the photoinduced charge carrier density. The first term on the RHS of Eq. 4 represents bimolecular recombination rate of photogenerated carriers, while the second term denotes monomolecular recombination rate. In addition, the recombination dynamics associated with the free charge carrier density (*n*) could be described by the following differential equation (Eq. 5) [35],

5 $$\frac{{{\text{d}}n}}{{{\text{d}}t}} = - k_{3} n^{3} - k_{2} n^{2} - k_{1} n$$

where *k*_3_, *k*_2_, and *k*_1_ represent the decay constants describing Auger recombination, the bimolecular recombination constant, and the rate for monomolecular processes such as trap-assisted charge recombination, respectively. Applying the above equations, the value of the bimolecular recombination rate was approximated to 1 × 10^−11^ cm^3^ s^−1^ in the baseline simulation model as reported for the rates of recombination ranging from 10^−11^ to 10^−9^ cm^3^ s^−1^ [36, 37]. Further, the interface recombination velocity (*S*) was evaluated by a numerical approach using the expression given below (Eq. 6).

6 $$S_{m} = \frac{{\mathop \smallint \nolimits_{\text{int}} R{\text{d}}x}}{m}$$

Here *R*, int, and *m* describe the recombination rate within the interface layer, the whole interface layer, and the concentration of holes and electrons, respectively. The values of *R* and *m* are estimable from the numerical solution, and the integral of *R* across the whole interface layer represents the total interface recombination as described in Eq. 3. The surface recombination velocities of electrons and holes at the top and bottom electrodes are estimated from Eq. 6 as 1 × 10^7^ cm s^−1^. The interface recombination losses in each device configuration could be regulated by introducing two thin interfacial defect layers hypothetically at the HTL/perovskite and ETL/perovskite interfaces [38, 39] as outlined in Table S1. This approach facilitates performance evaluation of the device as also determines the compatibility between theoretical and experimental data by interface engineering [39–42].

## Results and Discussions

The motivation to optimize efficiency and durability of PSCs emerged from the need to design a thermally stable perovskite with all-inorganic charge-transporting layers [43–46]. Using the codified AMPS-1D simulation tool, first-principle calculations were performed to find solutions for continuity and Poisson equations. The software was tweaked to simulate carrier transport in devices with single- and multi-junction formats. This approach allows the researcher to implement optimized structures for microelectronic, photovoltaic, or optoelectronic applications [30, 39, 41, 47].

**Fig. 2:**
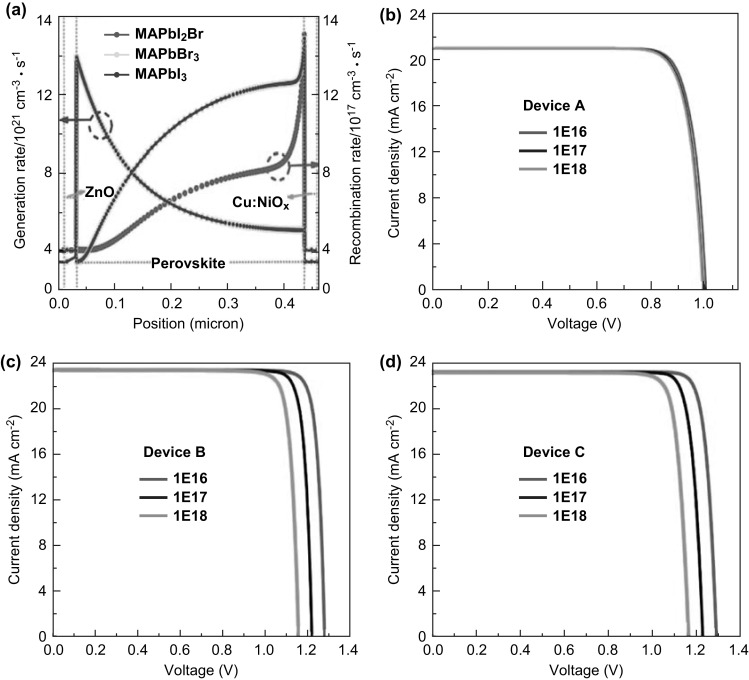
**a** Simulated values of carrier recombination and generation rates in MAPbX (MA = CH_3_NH_3_, X = I_3_, I_2_Br, or Br_3_) layers designed with Cu:NiO_*x*_ (HTM) and ZnO (ETM). **b**–**d** Simulated *J*–*V* characteristic curves for different defect densities in the absorber film

The modeling process and results have led to a better understanding of the fabrication process and perovskite material stability. The simulated current–voltage (*J*–*V*) characteristic curves of the devices A, B, and C are given in Fig. 1d. Device A (MAPbI_3_) showed the lowest PCE of 16.14%, open-circuit voltage (*V*_oc_) of 0.964 V, short-circuit current density (*J*_sc_) of 20.87 mA cm^−2^, and fill factor (FF) of 79%. Devices B (MAPbI_2_Br) and C (MAPbBr_3_) yielded the highest PCE values of 19.08 and 20.58%, *V*_oc_ of 1.098 and 1.107 V, *J*_sc_ of 22.53 and 22.99 mA cm^−2^, and FF of 79.45 and 81.67%, respectively. The simulated values of PCE of devices B and C are nearly consistent with the experimental value of 18.94% as reported by Kim et al. [46]. Notably, the realization of high *V*_oc_ and FF in devices B and C was attributed to the use of absorber materials with large energy bandgap and low mismatch between the work functions of Cu:NiO_*x*_ valence band, ZnO conduction band, and the perovskite layer [27, 48, 49]. Hole extraction from the perovskite layer is considered a key process in enhancing photovoltaic performance. The incorporation of halide (Br) in perovskite materials results in rapid hole extraction and low carrier recombination rate by precisely tuning the bandgap [46, 50]. As an added advantage, the long carrier diffusion lengths in such mixed-halide devices yield higher values of FF and PCE. With this in mind, we further investigated the photoelectric behavior of the best devices, namely MAPbI_2_Br and MAPbBr_3_ (in lieu of MAPbI_3_). It can be clearly seen in Fig. 2a that the recombination rate is reduced and the efficiency of charge carrier collection is improved by the application of Br-doped perovskites; the Cu:NiO_*x*_ (HTM) and ZnO (ETM) layers remained the same in both cases. A relatively small trap-assisted recombination was evident in MAPbI_2_Br. The above findings are consistent with the experimental results obtained using transient THz spectroscopy [51]. It is also in agreement with the studies of Liu et al. [6] and Liu and Kelly [28] who reported that the high degree of crystallinity in their perovskites helped to minimize the trap density within the material, thus lowering charge recombination and realizing high *V*_oc_ values. That a low recombination rate corresponds to high *V*_oc_ is referenced to the results of transient open-circuit voltage decay (OCVD) and intensity-modulated photovoltage spectroscopy (IMVS) measurements [52]. The enhanced device performance is also credited to low series resistance, efficient carrier transport, and absence of Schottky barrier in the charge carrier materials (HTM and ETM) and electrode interfaces [25, 53, 54]. In the following sections, we will discuss specific factors that influence the device performance, namely film thickness, defect density, operating temperature, and bandgap energy of perovskite materials. There is also evidence that the crystal structure and morphology play an important role in the optimization process and in further improvements of device performance.

### Effect of Defect Density

The number of defects in the absorber material is crucial to the device performance. By effectively managing the generation–recombination rate of the photogenerated carriers within the absorber layer, the *V*_oc_ of the device can be maximized. The simulated *J*–*V* curves in Fig. 2b–d illustrate that the defect states present in perovskite films cause a noticeable reduction in *V*_oc_, while its effect on *J*_sc_ is much less discernible; this is in accordance with experimental results related to Br-doped perovskites [46]. A high degree of crystallinity in experimentally formed perovskite layers results in low trap density, thus reducing charge recombination and realizing high *V*_oc_ values [6, 28]. It was earlier confirmed that the creation of a Br-rich layer within the tri-iodide perovskite structure enhanced moisture resistance, without sacrificing current density proving that the latter was less susceptible to defect density [55]. However, this resulted in a reduction in *V*_oc_. In our study, the *V*_oc_ of device B (MAPbI_2_Br) and device C (MAPbBr_3_) are higher than that of device A (MAPbI_3_) as shown in Fig. 2c–d. These results clearly establish that the relatively larger bandgap of the perovskite, especially in devices B and C (than in device A), has been effective in lowering the charge recombination rate and is responsible for improving charge extraction as depicted in Fig. 2a [6, 14, 28, 46, 56, 57].

### Effect of Active Layer Thickness

**Fig. 3:**
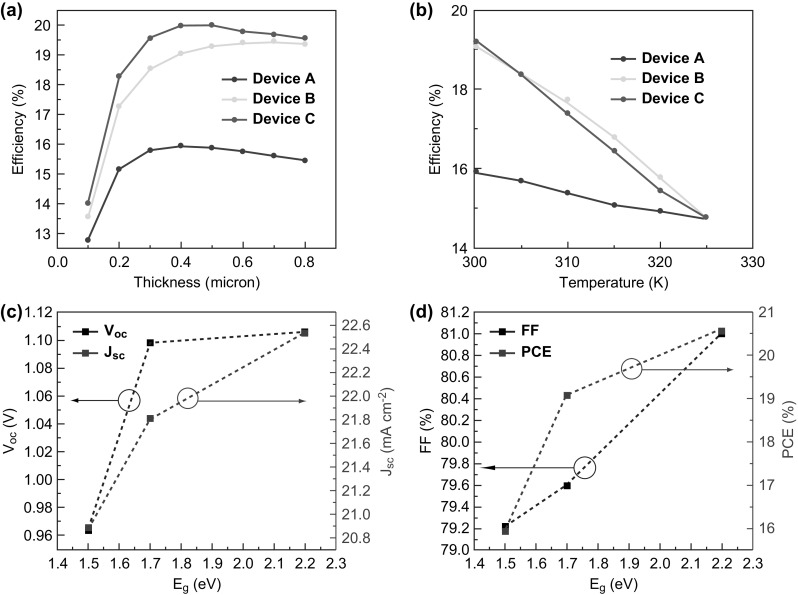
**a** Variation (simulated) of PCE obtained as a function of thickness of photoactive materials. **b** Variation (simulated) of PCE as a function of operating temperature. **c**, **d** Variation (simulated) of photovoltaic parameters as a function of bandgap energy in perovskite materials

The thickness of the photoactive perovskite layer also has a major role in improving the device performance. If the light-absorbing layer is too thin, the number of photons absorbed is small which will, in turn, give rise to low photocurrents; however, if a very thick perovskite layer is used, it results in low charge carrier extraction efficiency, and losses due to charge recombination become significant. Consequently, optimization of the absorber layer thickness assumes much importance in determining carrier generation and spectral response of the solar cells. Figure 3a shows the variation of PCE of the simulated devices as a function of the perovskite layer thickness. When the thickness was increased progressively in the range from 0.1 to 0.8 μm, the highest value of PCE was obtained for thicknesses of 0.4 for device A and 0.5 μm for devices B and C, respectively. The increase in efficiency is attributed to the increased number of electron–hole pairs generated in the photoactive layer. However, a decrease in efficiency at large thicknesses is due to increase in the recombination of charge carriers within the material before they reach the contact electrode. From the simulated results, which are summarized in Table 2, the thicknesses of 0.4 and 0.5 μm for perovskite layers were deduced as optimum values for achieving the highest PCE. Integrating the perovskite materials with suitable charge-transporting layers such as Cu:NiO_*x*_ (HTM) and ZnO (ETL), especially in the MAPbI_2_Br configuration, might serve as an effective method to collect charge carriers at low recombination rate using a specific absorber layer thickness(as illustrated in Fig. 2a). The process of accelerated hole extraction and improved carrier lifetime due to the Br concentration gradient in MAPb(I_1−*x*_Br_*x*_)_3_ has been verified by Kim et al. by observing photoluminescence properties in this material [14, 46, 58]. Additionally, the large photocurrents generated in these materials were attributed to the high absorption coefficient of the perovskite layers. We noticed a discrepancy between the simulated and experimental results at large values of thickness beyond the optimized thickness values (0.4 and 0.5 μm) which, we believe, is due to large variations in structure and thickness of the absorber layer in actual devices, which was also reported by Sum and Mathews [59]. Additionally, we found supporting evidence in the reports on planar heterojunction devices that employed higher layer thicknesses in the range of 0.4–0.8 μm for fabricating highly efficient devices. Besides, large carrier diffusion lengths of up to 1 μm have been observed in mixed-halide perovskites which also lends support to the claim that thicker absorber layers can be used [60]. Benefiting from the long carrier diffusion length, perovskite semiconductors possess long carrier diffusion length and a high charge collection efficiency [61]. Therefore, design of a thicker perovskite layer may serve as an effective solution to absorb more photons than that in solution-processed photovoltaic materials [62–65]. While our simulations have predicted the use of 0.4- and 0.5-μm-thick absorber layers for enhancing the device performance, available experimental data suggest that further increase in thickness may also help to achieve high values of PCE [61, 66, 67].

**Table 2:** Simulated photovoltaic characteristics of solar cells measured under standard AM1.5G illumination at 100 mW cm^−2^ irradiance

Device	*V*_oc_ (V)	*J*_sc_ (mA cm^−2^)	FF (%)	*η* (%)	Defect density (cm^−3^)	Optimized thickness (μm)
A	0.964	20.87	79	16.14	1 × 10^18^	0.4
B	1.098	22.99	79.45	19.08	1 × 10^18^	0.5
C	1.107	22.53	81.67	20.58	1 × 10^18^	0.5

### Effect of Operating Temperature

The performance of a PSC implicitly depends on its operating temperature. The standard test temperature (300 K) is generally used for performing simulations, but the operating temperatures in actual conditions are much higher. In this study, the simulation models considered the working temperature of 300 K as listed in Tables 1, 2 and S1. It is worthwhile to note that high temperature often leads to increased stress and deformation in devices which is likely to create more interfacial defects and cause poor inter-connectivity between the layers. Consequently, a high recombination rate prevails which is accompanied by reduction in carrier diffusion length and increase in series resistance [68, 69]. It has been previously reported that increase in temperature will significantly affect the electron and hole concentration, charge carrier mobility, material bandgap, and also device power conversion efficiency. This is illustrated by our simulation results as shown in Fig. 3b. However, it was found that the decrease in PCE can be controlled to a certain extent by incorporating a homogeneous layer of mixed-halide perovskite (Br-substituted perovskite). Kim et al. [27, 46] fabricated a thermally stable Br-doped perovskite solar cell with PCE of 18.94% under high humid conditions (40 − 90%) and retained its efficiency without encapsulation for 28 days. The use of inorganic HTLs and ETLs have established that, by optimizing thermal and chemical stability, improved performance can be realized in PSCs at lower material and processing cost [16, 17, 23, 25, 31, 70, 71].

### Effect of Energy Levels

Incident photon harvesting in a solar cell plays a large role in achieving high photovoltaic performance [72, 73]. Photoactive materials are broadly defined as those capable of responding to light or electromagnetic radiation. The optical absorption can be varied by tuning the bandgap energy of perovskite materials to achieve high device performance [14, 74]. The bandgap of MAPbX_3_ is considered to be one of the major factors that influence the magnitude of electronegativity difference between the metal cation and the anion in perovskite materials. An increase in the anion electronegativity is likely to diminish the covalent character of the halogen bond with Pb^2+^ ion and, consequently, result in an increase in the bandgap energy [14, 75]. Changes in composition with respect to the halide ion such as in MAPbI_3_, MAPbI_2_Br, and MAPbBr_3_ are likely to influence the optical properties of the perovskite materials as shown in Fig. S1. The simulated variation in the photovoltaic parameters obtained by changing the bandgap energy of the absorber material from 1.5 to 2.2 eV under optimized conditions is illustrated in Fig. 3c–d. It is clearly seen that with increasing bandgap energy, *V*_oc_ also increases in all cases. In a previous study related to the wide-bandgap perovskite (MAPb(I_1−*x*_Br_*x*_)_3_) solar cell, the results showed an increase in *V*_oc_ from 1.08 to 1.11 V and improvement of FF from 71 to 74% with thermally stable PCE of 18.94% [46]. The simulation results obtained in this study show an enhancement in FF and *J*_sc_ which are consistent with the energy band alignment of HTM and ETM with the absorber layer which can be seen in Figs. 1c and 2a, respectively [26]. These improvements have been attributed to increase in bandgap, reduced radiative recombination, and energy band matching [11, 23, 25, 27, 31, 46].

## Conclusion

In summary, the configurationally design of the perovskite solar cell MAPbX (MA = CH_3_NH_3_, X = I_3_, Br_3_, or I_2_Br) was systematically simulated using the simulation software wxAMPS which is an update of the popular solar cell simulation tool (AMPS; Analysis of Microelectronic and Photonic Structures). Various models were constructed with thin film stacks in the format, glass/FTO/Cu:NiO_*x*_/MAPbX/ZnO/Al. The shortcomings in each device configuration were overcome by introducing thin defect layers at the interfaces of HTL/absorber layer and ETL/absorber layer hypothetically. The simulated photovoltaic characteristics yielded the highest PCE value of 20.58% for the device C (MAPbBr_3_), 19.08% for device B (MAPbI_2_Br), and the lowest value of 16.14% for device A (MAPbI_3_). The simulation results also indicated that thicker perovskite layers realized higher values of PCE in relation to thinner ones due to enhanced photon harvesting. The *J*–*V* characteristics provided evidence that the defect states present in perovskite films cause a noticeable reduction in V_oc_. However, this has very nominal effect on *J*_sc_ value, particularly for the MAPbI_2_Br perovskite cell. The increase in bandgap energy is responsible for the enhanced photovoltaic parameters such as PCE, FF, *J*_sc_, and *V*_oc_. These findings are likely to serve as important inputs and guide to the design of new mixed-halide perovskite structures with Cu:NiO_*x*_-HTM and ZnO ETM for achieving high stability and low fabrication cost in perovskite solar cells.

## Electronic Supplementary Material

Below is the link to the electronic supplementary material.

Supplementary material 1 (PDF 128 kb)

## References

[1] Kojima A, Teshima K, Shirai Y, Miyasaka T (2009). Organometal halide perovskites as visible-light sensitizers for photovoltaic cells. J. Am. Chem. Soc..

[2] Song D, Cui P, Wang T, Wei D, Li M (2015). Managing carrier lifetime and doping property of lead halide perovskite by postannealing processes for highly efficient perovskite solar cells. J. Phys. Chem. C.

[3] Kim HS, Lee CR, Im JH, Lee KB, Moehl T (2012). Lead iodide perovskite sensitized all-solid-state submicron thin film mesoscopic solar cell with efficiency exceeding 9%. Sci. Rep..

[4] Lee MM, Teuscher J, Miyasaka T, Murakami TN, Snaith HJ (2012). Efficient hybrid solar cells based on meso-superstructured organometal halide perovskites. Science.

[5] Burschka J, Pellet N, Moon SJ, Humphry-Baker R, Gao P, Nazeeruddin MK, Gratzel M (2013). Sequential deposition as a route to high-performance perovskite-sensitized solar cells. Nature.

[6] Liu M, Johnston MB, Snaith HJ (2013). Efficient planar heterojunction perovskite solar cells by vapour deposition. Nature.

[7] Jeon NJ, Noh JH, Kim YC, Yang WS, Ryu S, Seok SI (2014). Solvent engineering for high-performance inorganic–organic hybrid perovskite solar cells. Nat. Mater..

[8] Jeon NJ, Noh JH, Yang WS, Kim YC, Ryu S, Seo J, Seok SI (2015). Compositional engineering of perovskite materials for high-performance solar cells. Nature.

[9] Yang WS, Noh JH, Jeon NJ, Kim YC, Ryu S, Seo J, Seok SI (2015). High-performance photovoltaic perovskite layers fabricated through intramolecular exchange. Science.

[10] Green MA, Emery K, Hishikawa Y, Warta W, Dunlop ED (2015). Solar cell efficiency tables (version 46). Prog. Photovolt: Res. Appl..

[11] Zhang F, Yang B, Mao X, Yang R, Jiang L (2017). Perovskite CH_3_NH_3_PbI_3−*x*_Br_*x*_ single crystals with charge-carrier lifetimes exceeding 260 μs. ACS Appl. Mater. Interfaces.

[12] McMeekin DP, Sadoughi G, Rehman W, Eperon GE, Saliba M (2016). A mixed-cation lead mixed-halide perovskite absorber for tandem solar cells. Science.

[13] Pellet N, Gao P, Gregori G, Yang TY, Nazeeruddin MK, Maier J, Gratzel M (2014). Mixed-organic-cation perovskite photovoltaics for enhanced solar-light harvesting. Angew. Chem. Int. Ed..

[14] Elseman AM, Shalan AE, Rashad MM, Hassan AM (2017). Experimental and simulation study for impact of different halides on the performance of planar perovskite solar cells. Mater. Sci. Semicond. Process..

[15] Zhang Z, Yue X, Wei D, Li M, Fu P, Xie B, Song D, Li Y (2015). DMSO-based PbI_2_ precursor with PbCl_2_ additive for highly efficient perovskite solar cells fabricated at low temperature. RSC Adv..

[16] Christians JA, Fung RCM, Kamat PV (2014). An inorganic hole conductor for organo-lead halide perovskite solar cells. Improved hole conductivity with copper iodide. J. Am. Chem. Soc..

[17] You J, Meng L, Song TB, Guo TF, Yang YM (2016). Improved air stability of perovskite solar cells via solution-processed metal oxide transport layers. Nat. Nanotechnol..

[18] Wei D, Ji J, Song D, Li M, Cui P (2017). A TiO_2_ embedded structure for perovskite solar cells with anomalous grain growth and effective electron extraction. J. Mater. Chem. A.

[19] Seo S, Park IJ, Kim M, Lee S, Bae C, Jung HS, Park NG, Kim JY, Shin H (2016). An ultra-thin, un-doped NiO hole transporting layer of highly efficient (16.4%) organic–inorganic hybrid perovskite solar cells. Nanoscale.

[20] Hu L, Peng J, Wang W, Xia Z, Yuan J (2014). Sequential deposition of CH_3_NH_3_PbI_3_ on planar NiO film for efficient planar perovskite solar cells. ACS Photonics.

[21] Lien H-T, Wong DP, Tsao N-H, Huang C-I, Su C, Chen K-H, Chen L-C (2014). Effect of copper oxide oxidation state on the polymer-based solar cell buffer layers. ACS Appl. Mater. Interfaces.

[22] Du X, Wang Y, Xia ZG, Zhou H (2015). Perovskite CH_3_NH_3_PbI_3_ heterojunction solar cells via ultrasonic spray deposition. Appl. Mech. Mater..

[23] Son M-K, Steier L, Schreier M, Mayer MT, Luo J, Grätzel M (2017). A copper nickel mixed oxide hole selective layer for Au-free transparent cuprous oxide photocathodes. Energy Environ. Sci..

[24] Sajid, Elseman AM, Ji J, Dou S, Huang P, Cui D, Wei M, Li M (2018). Novel hole transport layer of nickel oxide composite with carbon for high-performance perovskite solar cells. Chin. Phys..

[25] Jung JW, Chueh C-C, Jen AKY (2015). A low-temperature, solution-processable, Cu-doped nickel oxide hole-transporting layer via the combustion method for high-performance thin-film perovskite solar cells. Adv. Mater..

[26] Yue S, Liu K, Xu R, Li M, Azam M (2017). Efficacious engineering on charge extraction for realizing highly efficient perovskite solar cells. Energy Environ. Sci..

[27] Kim JH, Liang PW, Williams ST, Cho N, Chueh CC, Glaz MS, Ginger DS, Jen AK (2015). High-performance and environmentally stable planar heterojunction perovskite solar cells based on a solution-processed copper-doped nickel oxide hole-transporting layer. Adv. Mater..

[28] Liu D, Kelly TL (2014). Perovskite solar cells with a planar heterojunction structure prepared using room-temperature solution processing techniques. Nat. Photonics.

[29] Eperon GE, Burlakov VM, Docampo P, Goriely A, Snaith HJ (2014). Morphological control for high performance, solution-processed planar heterojunction perovskite solar cells. Adv. Funct. Mater..

[30] Liu F, Zhu J, Wei J, Li Y, Lv M, Yang S, Zhang B, Yao J, Dai S (2014). Numerical simulation: toward the design of high-efficiency planar perovskite solar cells. Appl. Phys. Lett..

[31] Sato K, Kim S, Komuro S, Zhao X (2016). Characteristics of Cu-doped amorphous NiO thin films formed by RF magnetron sputtering. Jpn. J. Appl. Phys..

[32] Hernández-Como N, Morales-Acevedo A (2010). Simulation of hetero-junction silicon solar cells with AMPS-1D. Sol. Energy Mater. Sol. Cells.

[33] Vurgaftman I, Meyer JR, Ram-Mohan LR (2001). Band parameters for III–V compound semiconductors and their alloys. J. Appl. Phys..

[34] Noel NK, Stranks SD, Abate A, Wehrenfennig C, Guarnera S (2014). Lead-free organic–inorganic tin halide perovskites for photovoltaic applications. Energy Environ. Sci..

[35] Wehrenfennig C, Liu M, Snaith HJ, Johnston MB, Herz LM (2014). Charge-carrier dynamics in vapour-deposited films of the organolead halide perovskite CH_3_NH_3_PbI_3−*x*_Cl_*x*_. Energy Environ. Sci..

[36] Y. Wang, Y. Liu, H. Zhou, Z. Xia, Simulation of perovskite solar cells with inorganic hole transporting materials, in *2015 IEEE 42nd Photovoltaic Specialist Conference (PVSC)* (2015). 10.1109/PVSC.2015.7355717

[37] Y. Wang, Z. Xia, Y. Liu, H. Zhou, Uniform perovskite photovoltaic thin films via ultrasonic spray assisted deposition method, in *2015 IEEE 42nd Photovoltaic Specialist Conference (PVSC)* (2015). 10.1109/PVSC.2015.7355719

[38] Cuiffi J, Benanti T, Nam WJ, Fonash S (2010). Modeling of bulk and bilayer organic heterojunction solar cells. Appl. Phys. Lett..

[39] Wang T, Chen J, Wu G, Li M (2016). Optimal design of efficient hole transporting layer free planar perovskite solar cell. Sci. Chin. Mater..

[40] Kemp KW, Labelle AJ, Thon SM, Ip AH, Kramer IJ, Hoogland S, Sargent EH (2013). Interface recombination in depleted heterojunction photovoltaics based on colloidal quantum dots. Adv. Energy Mater..

[41] Wang T, Chen J, Wu G, Song D, Li M (2017). Designing novel thin film polycrystalline solar cells for high efficiency: sandwich CIGS and heterojunction perovskite. J. Semicond..

[42] Liao P, Zhao X, Li G, Shen Y, Wang M (2018). A new method for fitting current–voltage curves of planar heterojunction perovskite solar cells. Nano-Micro Lett..

[43] Shalan AE, Mourtada Elseman A, Rasly M, Moharam MM, Lira-Cantu M, Rashad MM (2015). Concordantly fabricated heterojunction ZnO–TiO_2_ nanocomposite electrodes via a co-precipitation method for efficient stable quasi-solid-state dye-sensitized solar cells. RSC Adv..

[44] Song D, Wei D, Cui P, Li M, Duan Z (2016). Dual function interfacial layer for highly efficient and stable lead halide perovskite solar cells. J. Mater. Chem. A.

[45] Kim HS, Mora-Sero I, Gonzalez-Pedro V, Fabregat-Santiago F, Juarez-Perez EJ, Park NG, Bisquert J (2013). Mechanism of carrier accumulation in perovskite thin-absorber solar cells. Nat. Commun..

[46] Kim M-C, Kim BJ, Son D-Y, Park N-G, Jung HS, Choi M (2016). Observation of enhanced hole extraction in Br concentration gradient perovskite materials. Nano Lett..

[47] Boussettine AA, Belhadji Y, Benmansour A (2012). Modeling of tandem solar cell a-Si/a-SiGe using AMPS-1D program. Energy Proc..

[48] He J, Windstorm H, Hagfeldt A, Lindquist S-E (2000). Dye-sensitized nanostructured tandem cell-first demonstrated cell with a dye-sensitized photocathode. Sol. Energy Mater. Sol. Cells.

[49] Ryu S, Noh JH, Jeon NJ, Kim YC, Yang WS, Seo J, Seok SI (2014). Voltage output of efficient perovskite solar cells with high open-circuit voltage and fill factor. Energy Environ. Sci..

[50] Suarez B, Gonzalez-Pedro V, Ripolles TS, Sanchez RS, Otero L, Mora-Sero I (2014). Recombination study of combined halides (Cl, Br, I) perovskite solar cells. J. Phys. Chem. Lett..

[51] Wehrenfennig C, Eperon GE, Johnston MB, Snaith HJ, Herz LM (2014). High charge carrier mobilities and lifetimes in organolead trihalide perovskites. Adv. Mater..

[52] Bi D, Yang L, Boschloo G, Hagfeldt A, Johansson EM (2013). Effect of different hole transport materials on recombination in CH_3_NH_3_PbI_3_ perovskite-sensitized mesoscopic solar cells. J. Phys. Chem. Lett..

[53] Chen W, Wu Y, Yue Y, Liu J, Zhang W (2015). Efficient and stable large-area perovskite solar cells with inorganic charge extraction layers. Science.

[54] Chen W, Liu FZ, Feng XY, Djurišić AB, Chan WK, He ZB (2017). Cesium doped NiO_*x*_ as an efficient hole extraction layer for inverted planar perovskite solar cells. Adv. Energy Mater..

[55] Noh JH, Im SH, Heo JH, Mandal TN, Seok SI (2013). Chemical management for colorful, efficient, and stable inorganic–organic hybrid nanostructured solar cells. Nano Lett..

[56] Edri E, Kirmayer S, Cahen D, Hodes G (2013). High open-circuit voltage solar cells based on organic–inorganic lead bromide perovskite. J. Phys. Chem. Lett..

[57] Yin W-J, Shi T, Yan Y (2014). Unusual defect physics in CH_3_NH_3_PbI_3_ perovskite solar cell absorber. Appl. Phys. Lett..

[58] Cui P, Fu P, Wei D, Li M, Song D (2015). Reduced surface defects of organometallic perovskite by thermal annealing for highly efficient perovskite solar cells. RSC Adv..

[59] Sum TC, Mathews N (2014). Advancements in perovskite solar cells: photophysics behind the photovoltaics. Energy Environ. Sci..

[60] Liu D, Gangishetty MK, Kelly TL (2014). Effect of CH_3_NH_3_PbI_3_ thickness on device efficiency in planar heterojunction perovskite solar cells. J. Mater. Chem. A.

[61] Laban WA, Etgar L (2013). Depleted hole conductor-free lead halide iodide heterojunction solar cells. Energy Environ. Sci..

[62] Xing G, Mathews N, Sun S, Lim SS, Lam YM, Grätzel M, Mhaisalkar S, Sum TC (2013). Long-range balanced electron- and hole-transport lengths in organic-inorganic CH_3_NH_3_PbI_3_. Science.

[63] Cui P, Wei D, Ji J, Song D, Li Y (2017). Highly efficient electron-selective layer free perovskite solar cells by constructing effective *p*–*n*-heterojunction. Solar RRL.

[64] Song D, Ji J, Li Y, Li G, Li M (2016). Degradation of organometallic perovskite solar cells induced by trap states. Appl. Phys. Lett..

[65] Luo H, Lin X, Hou X, Pan L, Huang S, Chen X (2017). Efficient and air-stable planar perovskite solar cells formed on graphene-oxide-modified PEDOT:PSS hole transport layer. Nano-Micro Lett..

[66] Etgar L, Gao P, Xue Z, Peng Q, Chandiran AK, Liu B, Nazeeruddin MK, Grätzel M (2012). Mesoscopic CH_3_NH_3_PbI_3_/TiO_2_ heterojunction solar cells. J. Am. Chem. Soc..

[67] Zhang C, Luo Y, Chen X, Chen Y, Sun Z, Huang S (2016). Effective improvement of the photovoltaic performance of carbon-based perovskite solar cells by additional solvents. Nano-Micro Lett..

[68] Raga SR, Barea EM, Fabregat-Santiago F (2012). Analysis of the origin of open circuit voltage in dye solar cells. J. Phys. Chem. Lett..

[69] Shin B, Gunawan O, Zhu Y, Bojarczuk NA, Chey SJ, Guha S (2013). Thin film solar cell with 8.4% power conversion efficiency using an earth abundant Cu_2_ZnSnS_4_ absorber. Prog. Photovolt..

[70] Hou Y, Chen W, Baran D, Stubhan T, Luechinger NA (2016). Overcoming the interface losses in planar heterojunction perovskite-based solar cells. Adv. Mater..

[71] Liu Z, Sun B, Liu X, Han J, Ye H, Shi T, Tang Z, Liao G (2018). Efficient carbon-based CsPbBr_3_ inorganic perovskite solar cells by using Cu-phthalocyanine as hole transport material. Nano-Micro Lett..

[72] Zhang Z, Wei D, Xie B, Yue X, Li M, Song D, Li Y (2015). High reproducibility of perovskite solar cells via a complete spin-coating sequential solution deposition process. Sol. Energy.

[73] Zhang Z, Li M, Liu W, Yue X, Cui P, Wei D (2017). CH_3_NH_3_PbI_3_ converted from reactive magnetron sputtered PbO for large area perovskite solar cells. Sol. Energy Mater. Sol. Cells.

[74] Albrecht S, Saliba M, Correa-Baena J-P, Jäger K, Korte L, Hagfeldt A, Grätzel M, Rech B (2016). Towards optical optimization of planar monolithic perovskite/silicon-heterojunction tandem solar cells. J. Opt..

[75] Elseman AM, Shalan AE, Sajid S, Rashad MM, Hassan AM, Li M (2018). Copper substituted lead perovskites materials constructed with different halides for working (CH_3_NH_3_)_2_CuX_4_ based perovskite solar cells from experimental and theoretical view. ACS Appl. Mater. Interfaces..

