# Supplementary material for: Computational Study of Ternary Devices: Stable, Low-Cost, and Efficient Planar Perovskite Solar Cells
Source: Nanomicro Lett. 2018 May 17;10(3):51. doi: 10.1007/s40820-018-0205-5 (PMC6199104; doi:10.1007/s40820-018-0205-5)
Supplement: Supplementary file 1 — Supplementary material 1 (PDF 128 kb) [file 40820_2018_205_MOESM1_ESM.pdf]

Supporting Information for

# Computational Study of Ternary Devices: Highly Stable, Low Cost and Efficient Planar Perovskite Solar Cells

Sajid[1], Ahmed Mourtada Elseman [2], Jun Ji[1], Shangyi Dou[1], Dong Wei[1], Hao Huang[1], Peng Cui[1], Wenkang Xi[1], Lihua Chu[1], Yingfeng Li[1], Bing Jiang[1], Meicheng Li[1,]*

[1]State Key Laboratory of Alternate Electrical Power, System with Renewable Energy Sources, School of Renewable Energy, North China Electric Power University, Beijing 102206, People's Republic of China

[2]Electronic & Magnetic Materials Department, Advanced Materials Division, Central Metallurgical Research and Development Institute (CMRDI), Helwan, P.O.Box 87, Cairo 11421, Egypt

*Corresponding author. E-mail: mcli@ncepu.edu.cn (Meicheng Li); Tel: +86 10 6177 2951

## Supplementary Figure and Table

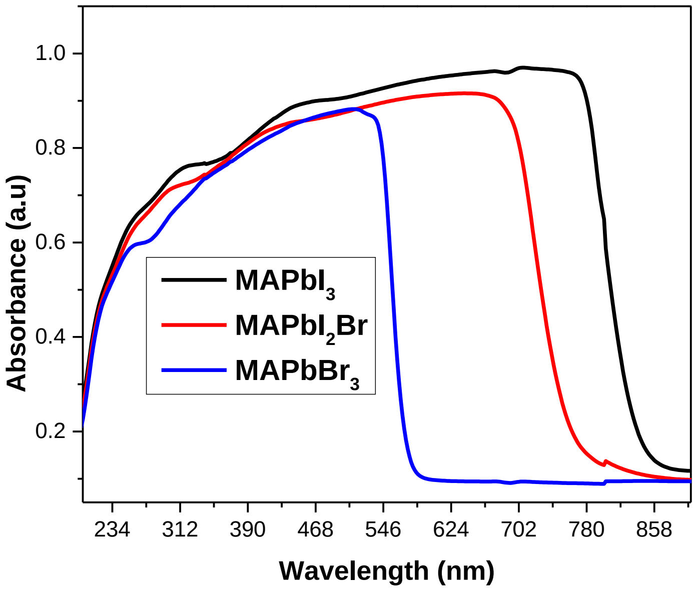


**Fig. S1** Absorbance spectra of the $MAPbI_3$, $MAPbI_2Br$ and $MAPbBr_3$ materials

**Table S1** Basic parameters for thin defect layers at the interfaces of ZnO/perovskite and Cu:$NiO_x$/perovskite

| **Parameters and units** | **ZnO/perovskite** | **Cu:$NiO_x$/perovskite** |
|---|---|---|
| Dielectric constant | 30 | 11 |
| Band gap (eV) | 1.5 | 3.6 |
| Electron affinity (eV) | 3.93 | 1.46 |
| Thickness (nm) | 2 | 2 |
| Electron and hole mobility($cm^2$/V/s) | 50, 50 | 0.5, 0.5 |
| Acceptor concentration ($cm^{-3}$) | $2.14\times10^{17}$ | $1.4\times10^{20}$ |
| Donor concentration ($cm^{-3}$) | 0 | 0 |
| Effective conduction and density ($cm^{-3}$) | $2.5\times10^{20}$ | $2\times10^{17}$ |
| Effective valence band density ($cm^{-3}$) | $2.5\times10^{20}$ | $1.1\times10^{19}$ |
| Characteristic energy for donor and acceptor-like tails (eV) | 0.015, 0.015 | 0.01, 0.01 |
| Band tail density of states (1/$cm^3$/eV) | $1\times10^{14}$ | $1\times10^{14}$ |
| Capture cross section for electrons and holes in donor tail states ($cm^2$) | $1\times10^{-15}$, $1\times10^{-17}$ | $1\times10^{-15}$, $1\times10^{-17}$ |
| Capture cross section for electrons and holes in acceptor tail states ($cm^2$) | $1\times10^{-17}$, $1\times10^{-15}$ | $1\times10^{-17}$, $1\times10^{-15}$ |
| Switch-over energy (eV) | 0.7 | 0.8 |
| Density of mid-gap acceptor and donor-like states ($cm^{-3}$ $eV^{-1}$) | $1\times10^{16}$ to $1\times10^{19}$ | $1\times10^{17}$ to $1\times10^{19}$ |
| Capture cross section of electrons and holes in donor mid-gap states ($cm^2$) | $1\times10^{-17}$, $1\times10^{-18}$ | $1\times10^{-16}$, $1\times10^{-17}$ |
| Capture cross section of electrons and holes in acceptor mid-gap states ($cm^2$) | $1\times10^{-18}$, $1\times10^{-17}$ | $1\times10^{-17}$, $1\times10^{-16}$ |
